# Increased microenvironment stiffness in damaged myofibers promotes myogenic progenitor cell proliferation

**DOI:** 10.1186/s13395-015-0030-1

**Published:** 2015-02-17

**Authors:** Frédéric Trensz, Fabrice Lucien, Vanessa Couture, Thomas Söllrald, Geneviève Drouin, André-Jean Rouleau, Michel Grandbois, Gregory Lacraz, Guillaume Grenier

**Affiliations:** Research Centre of the Centre Hospitalier de l’Université de Sherbrooke (CRCHUS), Université de Sherbrooke, Sherbrooke, QC Canada; Department of Electrical and Computer Engineering, Faculty of Engineering, Université de Sherbrooke, Sherbrooke, QC Canada; Department of Pharmacology, Faculty of Medicine, Université de Sherbrooke, Sherbrooke, QC Canada; Department of Orthopedic Surgery, Faculty of Medicine, Université de Sherbrooke, 3001–12th Avenue North, Sherbrooke, J1H 5N4, QC Canada; New address: Hubrecht Institute, University Medical Center Utrecht, Utrecht, The Netherlands

**Keywords:** Myofiber, Myogenic progenitor cells, Stiffness, Microenvironment

## Abstract

**Background:**

The stiffness of the myogenic stem cell microenvironment markedly influences the ability to regenerate tissue. We studied the effect of damaged myofibers on myogenic progenitor cell (MPC) proliferation and determined whether the structural integrity of the microenvironment contributes to phenotypic changes.

**Methods:**

Individual myofibers were isolated and cultured for 6 days. During this period, the cytoskeleton of myofibers and transcription factors regulating MPC differentiation were characterized by immunostaining. Atomic Force Microscopy (AFM) was performed to measure stiffness of cultured myofibers. Healthy and damaged myofibers, and their associated MPCs, were studied in skeletal muscle from dystrophic and tenotomy mouse models. MPCs were cultured on stiffness-tunable substrates, and their phenotypes were assessed by immunostaining of myogenic transcription factors.

**Results:**

We showed that individual myofibers tend to shrink or collapse when cultured *ex vivo* starting from day 1 and that this is associated with a marked increase in the number of proliferative MPCs (Pax7^+^MyoD^+^). The myofibers collapsed due to a loss of viability as shown by Evans blue dye uptake and the disorganization of their cytoskeletons. Interestingly, collapsed myofibers in *mdx* skeletal muscles were similar to damaged myofibers in that they lose their viability, have a disorganized cytoskeleton (actin and α-actinin), and display local MPC (MyoD^+^) proliferation at their periphery. In a tenotomy model that causes loss of muscle tension, the cytoskeletal disorganization of myofibers also correlated with the activation/proliferation of MPCs. A deeper analysis of collapsed myofibers revealed that they produce trophic factors that influence MPC proliferation. In addition, collapsed myofibers expressed several genes related to the basal lamina. Immunostaining revealed the presence of fibronectin in the basal lamina and the cytoplasm of damaged myofibers. Lastly, using atomic force microscopy (AFM), we showed that collapsed myofibers exhibit greater stiffness than intact myofibers. Growing MPCs on a 2-kPa polyacrylamide-based substrate, exempt of additional microenvironmental cues, recapitulated proliferation and reduced spontaneous differentiation compared to growth on a 0.5-kPa substrate.

**Conclusions:**

Our results support the notion that collapsed or damaged myofibers increase the structural stiffness of the satellite cell microenvironment, which in addition to other cues such as trophic factors and changes in extracellular matrix composition, promotes the proliferation and maintenance of MPCs, required for myofiber repair.

**Electronic supplementary material:**

The online version of this article (doi:10.1186/s13395-015-0030-1) contains supplementary material, which is available to authorized users.

## Background

Satellite cells are quiescent muscle stem cells that play a determinant role in myofiber turnover and postnatal regeneration [1]. Satellite cells are sequestered between the myofiber plasma membrane and the basal lamina [2], and their function is tightly regulated by numerous biochemical and cellular signals in their microenvironment or niche. These consist of subjacent myofiber anchoring; the extracellular matrix (ECM) that sequesters various growth factors; the neighboring cells from the stroma, including immune cells; and the environment of the circulatory system (for a recent review, see [3]). When favorable alterations occur in this complex microenvironment, satellite cells can activate and give rise to proliferative myogenic progenitor cells (MPCs) that repair myofibers, while others return to replenish the satellite cell pool.

Recent evidence has highlighted the importance of the mechanical forces exerted by the ECM in the microenvironment that can modulate the fate and function of satellite cells and MPCs [4-7]. More specifically, the elasticity/stiffness of the microenvironment can modulate *in vitro* myogenic stem cell renewal, proliferation, differentiation, and regenerative potential [7,8]. Boonen *et al.* showed that substrate elasticity strongly impacts MPC proliferation, while the protein coating influences their differentiation [7]. This is an important finding since it showed for the first time that the biomechanical microenvironment can influence primary MPC proliferation. Skeletal muscle stiffness is mainly determined by the composition of the ECM, notably fibrillar collagen [9,10]. First considered as a structural tissue that maintains the integrity of muscle fibers during contraction, it is now well established that the ECM plays a major role in the function of myogenic stem cell and muscle regeneration [11-13]. On the other hand, the mechanical properties of the ECM have an effect on force transmission by muscle fibers to the tendons. Excessive accumulation of ECM (fibrosis) and conditions in which muscle passive stiffness is heightened, such as during myopathy and aging, can impair satellite cell function and muscle regeneration [14-17].

When satellite cells are cultured on traditional plastic dishes, they permanently exit quiescence and acquire a high capacity to proliferate [18]. They concomitantly rapidly lose their self-renewal and regenerative potential, as shown by their limited contribution to muscle regeneration following transplantation [19,20]. In contrast, when satellite cells are directly transplanted following isolation or are transplanted in conjunction with subjacent myofibers (a microenvironment component), their potential for regeneration is maintained [21,22]. Interestingly, Gilbert *et al.* recently showed that culturing myoblasts on substrates of different stiffnesses can influence their self-renewal potential and their capacity to integrate tissues and replenish the stem cell pool when they are transplanted [8]. Other investigators have also shown that changes in ECM composition and/or quality can alter the biomechanical properties of skeletal muscles, which can in turn can modify the proliferation and differentiation potential of MPCs [7,23,24]. The loss of regenerative potential by satellite cells in aging, for example, has been attributed more to changes in their microenvironment than to intrinsic modifications [16,17,25-27]. This is why we hypothesized that structural changes in damaged myofibers may cause slight changes in the stiffness of the satellite cell microenvironment that may have an impact on their proliferative and differentiation capacities.

We report that individual myofibers cultured *in vitro* and damaged *in vivo* myofibers spontaneously shrink or collapse and lose their cytoskeletal organization concomitant with MPC activation/proliferation. We used atomic force microscopy (AFM)-based indentation experiments to show that collapse induced an increase in the stiffness of the satellite cell microenvironment that in turn promoted MPC proliferation. The present study provides new evidence that changes in the biomechanical microenvironment of the anatomical niche of myogenic stem cells have an impact on their behavior.

## Methods

### Animals

Three-month-old male wild-type (WT) C57Bl/6 (Charles River, Senneville, QC, Canada) mice, Myf5-nLacZ [28] (backcrossed in C57Bl/6) mice, and 8- to 12-month-old C57BL/10ScSn-Dmd/J (*mdx*) (The Jackson Laboratory, Bar Harbor, ME, USA) mice were raised in our animal colony. All animal experiments were approved by the Animal Ethics Committee of Université de Sherbrooke and were performed in accordance with Canadian Council on Animal Care guidelines (Protocol #133-14B). Surgeries were performed under anesthesia (isoflurane; Abbott Laboratories, Montreal, QC, Canada). To relieve postoperative pain, the mice were injected subcutaneously with 0.05 to 0.1 mg/kg of buprenorphine (Temgesic; Schering-Plough, Kenilworth, NJ, USA) 30 min prior to surgery.

### Myofiber and myoblast isolation and culture

Single myofibers from extensor digitorum longus (EDL) muscles were isolated by collagenase digestion as previously described [17], with slight modifications. Briefly, EDL muscles were digested with collagenase for 1 h at 37°C with frequent and gentle agitation. Myofibers were then harvested individually at room temperature using horse serum (HS)-coated Pasteur pipettes. Following several rinses with proliferative medium (Ham’s F10 supplemented with 20% fetal bovine serum (FBS; Hyclone, Life Technologies, Little Chalfont, Buckinghamshire, UK), 1% antibiotics, and 2.5 ng/ml of bFGF), the myofibers were incubated again with collagenase for 10 min and were then transferred to proliferative medium and incubated at 37°C in a 5% CO_2_ humidified incubator for 6 days. Intact and collapsed myofibers were either fixed in 4% PFA for 10 min, rinsed in PBS, and stored at 4°C for immunofluorescence studies or were triturated with HS-coated Pasteur pipettes to isolate peripheral MPCs (or myoblasts). Isolated myoblasts were then enriched and were amplified by four passages in proliferative medium in collagen-coated petri dishes.

### Quantitative PCR

Quantitative PCR (qPCR) was performed as previously described [25,29]. Total RNA was extracted using TRIzol® (Invitrogen, Burlington, ON, Canada) according to the manufacturer’s instructions. The RNA was precipitated with isopropanol and 1 μg of glycogen, rinsed with ethanol, and resuspended in RNAse-free water. The RNA was reverse transcribed using RT Superscript II kits (Invitrogen). The qPCR reactions were prepared with PerfeCTa® SYBR® Green SuperMix (Quanta Biosciences, Gaithersburg, MD, USA). The samples were then placed in a RotorGene 6000 (Corbett Robotics, San Francisco, CA, USA). The qPCR conditions were set as follows: 10 min at 95°C; 40 cycles of 40 s at 95°C and 40 s at 56°C. The results were analyzed using the 2^−ΔΔCT^ relative quantification method normalized to the 18S housekeeping gene (NR_003286; forward: AGG AAT TGA CGG AAG GGC AC; and reverse: CGA CAT CTA AGG GCA TCA CA). Commercial primers were used for fibronectin (Mm_Fn1_1_SG), collagen IV (Mm_Fn1_1_SG), laminin (Mm_Lama1_1_SG), collagen VI (Mm_Col6a1_1_SG), and IGF-1 (Mm_Igf1_1_SG), all from Qiagen (QuantiTect Primer Assay, Venlo, Limburg, Netherlands).

### Preparation of conditioned media from myofibers

Following EDL muscle digestion, 100 intact and 100 collapsed myofibers were harvested, transferred into separate 6-cm petri dishes containing 2.5 ml of myoblast growth medium w/o bFGF (Ham’s F10 supplemented with 20% fetal bovine serum [FBS; Hyclone], and 1% antibiotic), and cultured at 37°C in a 5% CO_2_ humidified incubator. After 3 days, conditioned media were collected, centrifuged at 1,200 g (15 min, 4°C), and filtered (0.45 μm) and stored at 4°C. Conditioned media were used within 24 h.

Cultured primary myoblasts were passaged and plated at 2,000 cells/cm^2^ in 48-well plates that contained myoblast growth medium with bFGF. After 24 h, medium was replaced by conditioned media (250 μl), and cells were cultured for 2 days. For proliferation quantification, cells were fixed in 4% PFA for 10 min and nuclei stained with DAPI. Five 10× pictures per well (four wells/preparation) were randomly taken using an inverted fluorescent microscope (Leica DM-IRE2; Nikon Instruments Inc., Chicago, IL, USA) and were used to count cell numbers.

### Tenotomy

The tibialis anterior (TA) muscles of WT and Myf5-nLacZ mice were exposed by a 1-cm incision in the skin and fascia. The right TA distal tendon was sectioned, and the incision was closed using three stitches. After 10 days, the mice were euthanized, and the TA muscles were harvested for histological studies.

### Determination of permeable myofibers *in vivo*

To demonstrate the presence of damaged myofibers *in vivo*, WT and *mdx* mice were i.p. injected with 1% (w/v) Evans blue dye (EBD) (Sigma-Aldrich, Oakville, ON, Canada) in sterile PBS as described previously [30]. TA and biceps femoris (BF) muscles were harvested 24 h after the EBD injection.

### Histology and immunofluorescence

Harvested muscles were immersed in successive PBS baths containing stepped concentrations of sucrose (5%, 15%, and 30%). The TA and BF muscles were then embedded in OCT:30% sucrose (O.C.T.™; Torrance, CA, USA), flash-frozen in isopentane chilled in liquid nitrogen, and stored at −80°C until analyzed. Ten-micrometer sections were cut beginning at the midpoint of the muscles using a cryostat (CM1850; Leica, Canada). To evaluate viability, intact and collapsed myofibers freshly isolated from the EDL muscles of WT mice or after 6 days of culture were immersed in 5% EBD for 5 min and were then rinsed with PBS.

For the immunofluorescence experiments, intact and collapsed myofibers isolated from the EDL muscles of WT mice, cell preparations, or 10-μm-thick TA and BF muscle sections were fixed with 4% PFA at 4°C for 10 min and were permeabilized in PBS containing 10% goat serum, 1% BSA, and 0.2% Triton®X-100 (Sigma-Aldrich). The cells, myofibers, and tissue sections were incubated with mouse anti-Pax7 (1:2; DSHB, Iowa City, IA, USA), mouse anti-myogenin (1:3; DSHB), mouse anti-α-actinin (1:1,000; Sigma), rabbit anti-MyoD (1:200; C-20, Santa Cruz, CA, USA), rabbit anti-fibronectin (1:100; Millipore, Billerica, MA, USA), or rabbit anti-Ki67 (1:100; Abcam, Cambridge, UK) primary antibodies or with Alexa 488-conjugated phalloidin (1:1,000; Invitrogen, Canada). After several rinses in PBS-Tween, the samples were incubated with Alexa Fluor® 488-conjugated goat anti-mouse IgG (1:1,000) secondary antibody (Invitrogen, Canada). Samples in which the primary antibodies were omitted served as controls. Cell nuclei were labeled with DAPI reagent (Sigma-Aldrich). Following MyoD staining, the BF sections were exposed to light for 48 h in PBS to bleach the fluorescence. An Axioskop 2 phase-contrast/epifluorescence microscope (Carl Zeiss, Inc., Dublin, CA, USA) was used to examine the staining of the tissue sections and myofibers. A Leica DM-IRE2 inverted microscope (Nikon Instruments Inc., USA) was used to examine the cell preparations. The photomicrographs were processed using Image Pro (Media Cybernetics, Rockville, MD, USA) and Simple PCI (Hamamatsu Corporation, Bridgewater, NJ, USA) software.

For X-gal staining, intact and collapsed myofibers isolated from the EDL muscles of Myf5-nLacZ mice as well as the TA muscle sections were fixed with 4% PFA at 4°C for 10 or 60 min. The preparations were then rinsed in 0.1 M phosphate buffer (pH 7.3) containing 2 mM MgCl_2_ and were incubated for a further 30 min in a detergent solution containing 0.1 M phosphate buffer (pH 7.3), 2 mM MgCl_2_, 5 mM potassium ferricyanide, 5 mM potassium ferrocyanide, 0.01% sodium deoxycholate, and 0.02% Nonidet P40 (Sigma-Aldrich). The preparations were then incubated in rinsing solution supplemented with 1 mg/ml X-gal reagent (Wisent Inc., Saint Jean-Baptiste, QC, Canada) overnight at 37°C. When the preparations were sufficiently stained, they were rinsed in PBS.

### Quantification of myofiber stiffness by atomic force microscopy

Myofibers were isolated from the EDL muscles of WT mice using the procedure described above. Intact myofibers were either used directly for the stiffness evaluation or were incubated in proliferative medium for 6 days. Intact and collapsed myofibers were transferred into six-well plates containing a piece of adhesive tape in the bottom of each well. The plates were centrifuged at 400 g for 10 min at room temperature to attach the myofibers to the tape. The pieces of tape with the attached myofibers were then transferred into petri dishes and were immersed in PBS for the stiffness evaluation. To evaluate differentiated myogenic cell stiffness, the C2C12 differentiation medium was replaced with PBS. Stiffness was quantified with an AFM-based indentation approach using a custom-built force measurement device similar to the AFM setup described elsewhere [31]. Our setup was mounted on an inverted microscope (Observer Z1; Carl Zeiss, Germany) that allowed the precise positioning of the cantilever tip above the myofiber. Prior to each experiment, the deflection sensitivity was determined in PBS in a plastic petri dish containing no myofibers or myotubes. The nominal spring constant of the silicon nitride cantilever (provided by the manufacturer) was used to convert the photodiode signal into a force value (k_nom_ = 0.05 N/m, MLCT; Bruker AFM Probes, Camarillo, CA, USA). The tip of the cantilever was positioned in the middle of each myofiber or myotube, and three force-indentation curves were collected. The data was analyzed using an in-house code in Matlab (MathWorks, Natick, MA, USA) based on the algorithm of Crick *et al.* [32]. A stepwise Young’s modulus was extracted from each force-indentation curve using a modified Hertz model for a four-sided pyramidal indenter [33]:

$$ F=\frac{E}{1-{\nu}^2}\;\frac{\;\left( tan\;\alpha \right)}{\sqrt{2}}{\delta}^2, $$

where *F* is the indentation force, *E* the Young’s modulus, *ν* the Poisson’s ratio (set to 0.5 for isotropic incompressible materials), *δ* the indentation, and *α* the face angle of the pyramid (17.5° for our cantilever). The mean of three Young’s modulus values was calculated for each myofiber or myotube and was used as an expression of stiffness.

### Synthesis of stiffness-tunable polyacrylamide gels

Stiffness-tunable substrates were synthesized using the method described by Tse and Engler [34]. Briefly, 25-mm^2^ round glass coverslips were immersed in 0.1 M NaOH and were placed on a 80°C hot plate until the NaOH evaporated. The coverslips were coated with 3-aminopropyltriethoxysilane (APES), which allows gel adhesion, in a nitrogen-filled tent. The coverslips were rinsed several times with distilled water and then with 0.5% glutaraldehyde in PBS. Glass slides were treated in parallel with dichlorodimethylsilane (DCDMS) for 5 min. Excess DCDMS was wiped off, and the glass slides were rinsed with distilled water. Gel substrates (0.5 and 2 kPa) were synthesized by mixing 0.75 ml (7.5% final concentration) of a 40% acrylamide solution and 0.3% ml (3.0% final concentration) of a 2% bis-acrylamide stock solution with 8.95 ml of distilled deionized water, 1 ml (10% final concentration) of a 40% acrylamide stock solution, and 0.5 ml (5% final concentration) of a 2% bis-acrylamide stock solution with 8.5 ml of distilled deionized water, respectively. The mixtures were degassed under vacuum for 15 min. The substrates were polymerized by adding ammonium persulfate (1:100) and Temed (1:1,000). A 25 μl of gel solution was quickly sandwiched between a pre-treated glass slide and a pre-treated coverslip. The polymerized gels were stored at 4°C in 40% ethanol until used. The day before they were used, the gel sandwiches were rinsed in PBS and were collagen coated to improve cell adhesion. MPCs (1,750 cells/cm^2^) were plated on the sandwiches and were cultured in proliferative medium. Proliferation was calculated using a Neubauer hemacytometer.

### Measurement of cell adherence on stiffness-tunable substrate

To determine whether polyacrylamide interferes with cell adhesion, MPC proliferation was halted by a 2-h treatment with 20 μg/ml of mitomycin-C (Cayman Chemical Company, Ann Arbor, MI, USA). The cells were then rinsed with PBS, trypsinized, centrifuged, resuspended in fresh medium, and plated at 10,000 cells/cm^2^ on substrates of different stiffnesses. After 48 h, the cells were fixed with 4% PFA, stained with DAPI, and counted.

### Statistics

All data are expressed as means ± SEM. Unpaired student *t-*tests were used to assess statistical significance between groups of fresh and cultured myofibers. A paired *t-*test was used to compare tenotomized muscles with sham-treated contralateral muscles. An F-test of equality of variance was used to compare the periodicity and width of the myofibers. A Kruskal-Wallis test was used to analyze multiple groups. *P* < 0.05 was considered to be statistically significant. Statistical values were obtained using GraphPad Prism 6.0 software™.

## Results

### Culturing individual myofibers induces collapse-associated MPC proliferation

**Figure 1:**
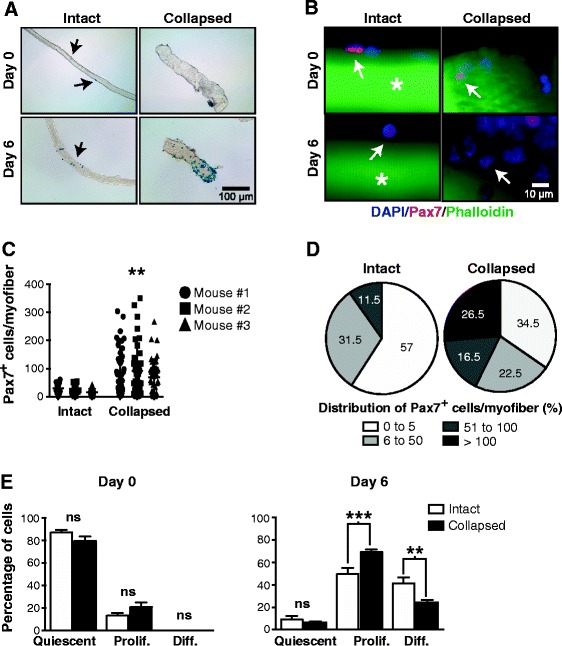
**Collapsed individual myofibers induce MPC proliferation. (A)** Representative X-gal staining of intact and collapsed myofibers isolated from the EDL muscles of Myf5-nLacZ mice after 0 and 6 days of culture. Arrows indicate Myf5^+^ cells. **(B)** Representative photomicrographs of intact and collapsed myofibers cultured for 0 and 6 days and immunostained with a Pax7-specific antibody (red) and stained with fluorochrome-conjugated phalloidin and DAPI to reveal actin filaments (F-actin, green) and nuclei (blue), respectively. The arrows indicate MPCs (Pax7^+^ cells). **(C)** Scatter dot plot showing the number of Pax7^+^ cells per intact or collapsed myofiber after 6 days of culture. Myofibers from the EDL muscles of three adult WT mice were immunolabeled with a Pax7-specific antibody. Nuclei were stained with DAPI (*n* = 0 to 43 myofibers). **P = 0.0024 versus intact myofibers. **(D)** Percentage of the distribution of the number of Pax7^+^ cells per intact and collapsed myofiber after 6 days of culture. **(E)** Bar graphs representing the proportion of MPCs in various differentiation states for intact or collapsed myofibers after 0 and 6 days of culture. Following immunostaining of MPCs with Pax7 and MyoD, the differentiation states were defined in terms of combinations of positive staining: quiescent (Pax7^+^MyoD^−^), proliferative (Pax7^+^MyoD^+^), and differentiating (Pax7^−^MyoD^+^). The results are from three independent experiments. Results are expressed as means ± SEM. **P < 0.01; ***P < 0.0001 versus intact and freshly isolated myofibers.

To better understand the muscle regenerative process, we studied MPC regulation using individual myofibers. We cultured individual myofibers isolated from Myf5-nLacZ mouse EDL muscles for 6 days in proliferative medium. Since these mice express nuclear β-galactosidase (β-gal) under the control of the *Myf5* promoter, which encodes a MPC-specific transcription factor [28], MPCs were localized by X-gal staining. Unlike intact myofibers, we observed a dramatic increase in X-gal staining in collapsed myofibers, which was associated with Myf5^+^ MPCs, after 6 days (Figure 1A). Interestingly, some myofibers shrank or collapsed, becoming larger and shorter during the first few days of culture (Figure 1A, collapsed myofibers after 6 days). We then determined the time-lapse increase in internal tension over 6 days that led to the collapse phenotype of the cultured myofibers. Intact myofibers collapsed by nearly 50% after 24 h in culture and by more than 80% after 6 days (Additional file 1: Figure S1). Unlike intact myofibers, collapsed myofibers displayed a marked increase in cellular budding on their periphery after 6 days in culture, which is a hallmark of MPC proliferation. In addition, intact myofibers retained their typical striated structure over the entire 6 days of culture, while the collapsed myofibers exhibited impaired internal organization of their actin filaments, as shown by phalloidin staining (Figure 1B). We quantified the number of MPCs in cultured myofibers after 6 days by immunostaining with an antibody directed against the myogenic marker Pax7. Only myofibers containing MPCs (Pax7^+^) were taken into consideration. As expected, based on the cellular budding, there was a significant increase in the number of MPCs in collapsed myofibers compared to intact myofibers (3.5-fold, *P* = 0.0024; Figure 1C). The number of MPCs in individual myofibers from a given mouse was highly variable and ranged from 0 to more than 300 cells after 6 days (Figure 1C). It is noteworthy that 26.5% of the collapsed myofibers had more than 100 Pax7^+^ cells per myofiber after 6 days of culture compared to only 11.5% of the intact myofibers (Figure 1D). These results showed that, following isolation and culture, most myofibers develop a shrunken or collapsed morphology concomitant with an increase in MPC proliferation.

The phenotypes of MPCs in freshly isolated and cultured myofibers were further characterized by Pax7 and MyoD staining, which makes it possible to determine whether the cells are in a quiescent (Pax7^+^MyoD^−^), proliferative (Pax7^+^MyoD^+^), or differentiating (Pax7^−^MyoD^+^) state (Figure 1E) [3,25,35,36]. Our results showed that both freshly isolated intact and collapsed myofibers display a similar proportion of quiescent, proliferative, and differentiating cells, with the quiescent state predominating (more than 90%). Proliferative and differentiating cells predominated in cultured myofibers, while many fewer cells were in the quiescent state. As expected, the proportion of proliferative cells was significantly higher in collapsed myofibers than in intact myofibers (*P* < 0.0006). As such, the proportion of differentiating cells was also significantly lower (*P* < 0.006), suggesting that the collapse of myofibers promotes MPC proliferation.

### The collapse of myofibers induces the proliferation of myogenic progenitor cells in damaged muscle

**Figure 2:**
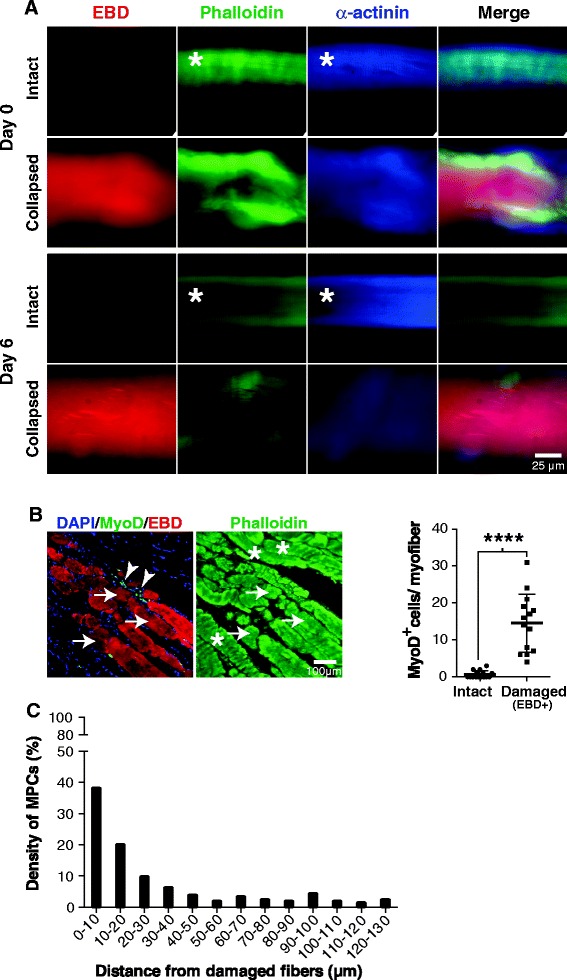
**The collapse of myofibers induces the proliferation of myogenic progenitor cells in damaged muscle** ***in vivo*** **. (A)** Representative photomicrographs of freshly isolated intact myofibers and collapsed myofibers from a C57Bl/6 WT mouse (*n* = 3 to 4). After 0 or 6 days of culture, the myofibers were incubated with Evans blue dye (EBD) to stain collapsed myofibers. The myofibers were then immunostained with fluorochrome-conjugated phalloidin to visualize F-actin and with anti-α-actinin to visualize both cytoskeleton proteins. Unlike collapsed myofibers, the intact myofibers displayed periodicity with the phalloidin and α-actinin staining in both the 0- and 6-day cultures. **(B)** Representative image of BF muscle longitudinal sections from 8- to 12-month-old *mdx* mice that had received an intraperitoneal injection of EBD 24 h prior to euthanasia (*left* panels). The sections were stained with anti-MyoD (green) and DAPI (blue). The actin cytoskeleton was stained with phalloidin (green) and DAPI (blue). Damaged myofibers were positive for EBD^+^ (red) whereas intact myofibers were EBD^−^ (*n* = 15 for intact myofibers and *n* = 4 for damaged myofibers; *n* = 3 mice). Arrows indicate degenerative EBD^+^ myofibers, and arrowheads indicate MyoD^+^ cells (activated/proliferative MPCs). Asterisks indicate intact myofibers with the striated pattern of actin. Scatter dot plot showing the number of MyoD^+^ cells per myofiber (*right* panel). Overlay images were mounted in Image Pro and were used to identify MyoD^+^ cells. ****P < 0.00001 versus control (intact myofibers). **(C)** Bar graph representing the distance of MPCs from EBD^+^ myofibers. After counting the number of cells, distance measurements were obtained by counting the number of MyoD^+^ cells in four different regions per muscle (*n* = 6 muscles). All results are expressed as means ± SEM.

To further characterize the collapsed myofibers, we investigated their viability using Evan Blue dye (EBD), which penetrates non-viable and degenerative myofibers [30]. While the intact myofibers remained unstained over the 6 days of culture, the collapsed myofibers were stained blue (EBD^+^) (Figure 2A). The cytoskeletal organization of intact and collapsed myofibers was further characterized using α-actinin, a cytoskeletal protein that forms a lattice-like structure and stabilizes the muscle contractile apparatus [37]. Our results showed that both freshly isolated (day 0) and cultured (day 6) EBD^−^ myofibers had an organized cytoskeleton, as indicated by the periodicity of the phalloidin-stained F-actin and α-actinin, while EBD^+^ myofibers were completely disorganized and lacked the characteristic periodicity due the loss of muscle Z-lines (Figure 2A).

Given that dystrophic muscles are characterized by collapsed myofibers and a disorganized cytoskeleton [38-42], we determined whether myofiber collapse was also associated with local proliferation of MPCs *in vivo*. We injected EBD into *mdx* mice, a model of Duchenne muscular dystrophy characterized by a high number of degenerative myofibers [38]. Histological analyses revealed the presence of sparse EBD^+^ myofibers throughout the muscles (Figure 2B). In addition, we showed that MyoD^+^ cells are approximately 15 times more numerous near EBD^+^ myofibers than near unstained myofibers (Figure 2B) and that 80% of them are clustered within 30 μm of the EBD^+^ myofibers (Figure 2C), indicating that local cues favor MPC proliferation.

**Figure 3:**
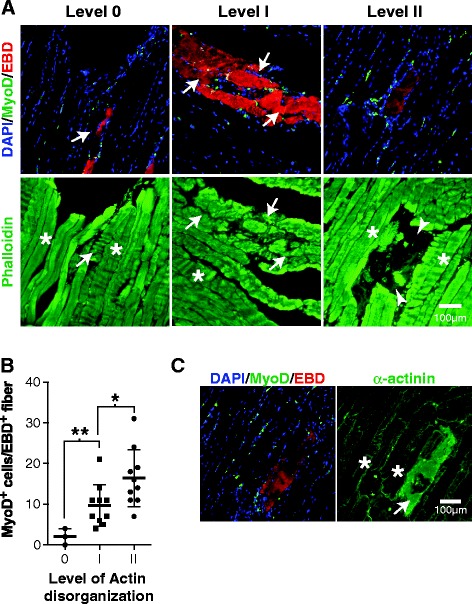
**Collapsed myofibers are degenerative cells with a disorganized cytoskeleton. (A)** Representative photomicrographs of BF muscle sections showing different levels of actin organization. Level 0 corresponds to organized actin similar to normal EBD^−^ myofibers, level I corresponds to actin disorganization (loss of periodicity and partial fragmentation), and level II corresponds to advanced fragmentation and/or loss of staining. Arrows indicate EBD^+^ myofibers, and asterisks indicate intact myofibers. Arrowheads indicate the loss of phalloidin staining (level II). **(B)** Scatter dot plot quantifying the relative density of MyoD^+^ cells in terms of actin disorganization. These results were obtained from four sections per muscle of BF (*n* = 3 muscles). **(C)** Representative images of BF tissue sections showing α-actinin impairment in damaged myofibers compared to intact myofibers. BF sections were stained with anti-α-actinin (green). Nuclei and MPCs were stained with DAPI and anti-MyoD, respectively. The asterisks and arrow indicate normal and impaired α-actinin patterns, respectively. *P < 0.05, **P < 0.01. All results are expressed as means ± SEM.

Since collapsed myofibers displayed obvious cytoskeletal disorganization (see Figures 1B and 2A), we further characterized the cytoskeletons of damaged (EBD^+^) *mdx* myofibers. Our observations showed that the F-actin in EBD^+^ myofibers, as revealed by phalloidin staining (Figure 3A), can be classified into three types of organization: 0, organization similar to normal EBD^−^ myofibers; I, disorganization (loss of periodicity); and II, fragmented and/or absence of staining. Based on this classification scheme, we were able to show that there is a significant correlation between the state of actin organization and the number of MyoD^+^ cells associated with EBD^+^ myofibers (Figure 3B), i.e., the higher the level of disorganization the higher the number of MyoD^+^ cells. Interestingly, α-actinin staining showed that 100% of EBD^+^ myofibers display disorganized α-actinin (*n* = 40), as revealed by the loss of periodicity due the loss of muscle Z-lines (Figure 3C). While phalloidin staining (F-actin) is suitable for categorizing the degree of myofiber degeneration (and MPC proliferation), α-actinin may be useful for detecting earlier phases in which F-actin is not yet disorganized.

**Figure 4:**
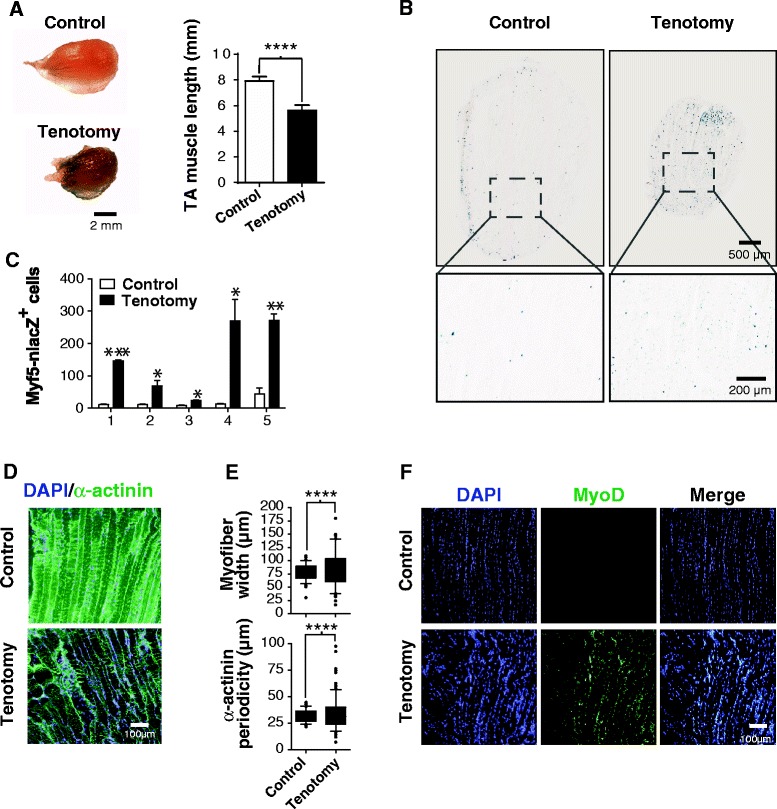
**α** **-Actinin disorganization in myofibers from tenotomized muscle correlates with scattered MPC proliferation. (A)** Representative photomicrographs of X-gal-stained control and tenotomized TA muscles 10 days post-tenotomy. Note the shortening and stronger blue staining of the tenotomized TA muscle compared to the contralateral control muscle, indicating the presence of a large number of Myf5-nLacZ^+^ MPCs in the tenotomized TA muscle. **(B)** Representative images of X-gal-stained longitudinal sections from the same regions of TA muscles from Myf5-nlacZ mice. The number of MPCs was quantified by counting the number of lacZ-expressing MPCs in the tenotomized TA and contralateral control muscles (three sections per muscle). **(C)** Histograms showing the total number of quiescent and proliferating MPCs (Myf5^+^ cells). These results were obtained by counting the number of Myf5^+^ cells per field in the same regions of the tenotomized TA and contralateral muscles (three sections per muscle) (*n* = 5 mice). **(D)** Representative photomicrographs of a tenotomized TA muscle showing α-actinin (green) disorganization (*top* panels) and MyoD^+^ cells (green) (*bottom* panels). **(E)** Box-and-whisker plot (90th percentile) showing the average width (*left* panel) and periodicity (*right* panel) of myofibers in tenotomized TA muscles compared to intact contralateral control muscles (*n* = 38 for intact TA muscles and *n* = 66 for tenotomized TA muscles; *n* = 4 mice). An F-test for equality of variances was used to determine the distribution of measurements in the two groups (intact versus tenotomized muscles) **(F)** Representative photomicrographs of MyoD immunostaining showing a large number of MyoD^+^ cells scattered among the disorganized myofibers of tenotomized muscles. *P < 0.05, **P < 0.01, ***P < 0.0001, and ****P < 0.00001 versus the contralateral control muscle. All results are expressed as means ± SEM.

To confirm that the induction of MPC proliferation was caused by the loss of tension in myofibers, we tenotomized the distal tendons of the TA muscles of Myf5-nlacZ mice. After 10 days, tenotomized and contralateral control TA muscles were isolated and were stained with X-gal. Gross observations revealed a significant retraction of the tenotomized muscles (approximately 30%; *P* < 0.0001, *n* = 5 mice) with respect to the contralateral control TA muscles (Figure 4A). Moreover, stronger X-gal staining was observed in the tenotomized TA muscles (Figure 4A). Histological sections were used to quantity MPC proliferation by counting the number of lacZ^+^ cells (MPCs) in the same regions of the muscles (Figure 4B). As expected, there was a significant 3- to 20-fold increase (*P* = 0.0313 to *P* < 0.0001; Figure 4C) in the number of proliferating and quiescent MPCs in the tenotomized TA muscles compared to the contralateral control muscles, indicating that a loss of tension also induces MPC proliferation *in vivo*.

Given their robust retraction over time, we looked at whether the myofibers from these muscle preparations showed evidence of a loss of viability and/or cytoskeletal disorganization compared to the contralateral control muscles. Surprisingly, there were virtually no EBD^+^ myofibers in the muscle preparations. However, immunohistological analyses showed that myofibers from the control muscles had an organized cytoskeleton, as revealed by the periodicity of the α-actinin (Figure 4D). On the other hand, myofibers from the tenotomized muscles appeared to have a less organized pattern (Figure 4D). There was a significant difference in the width and periodicity of the α-actinin staining of the myofibers in the tenotomized muscles compared to the contralateral muscles (Figure 4E). The myofibers in the tenotomized muscles also contained a larger number of MyoD^+^ cells (Figure 4F).

### The microenvironment of collapsed myofibers is associated with alterations in the ECM and trophic factors

**Figure 5:**
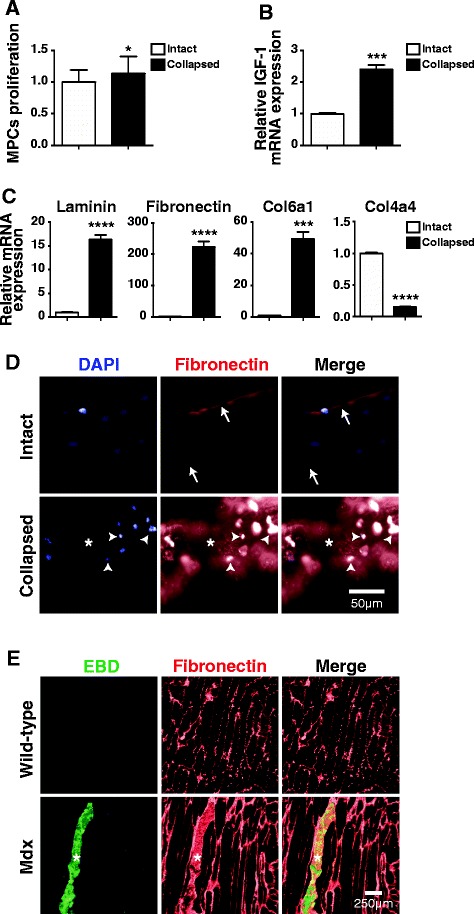
**The microenvironment of collapsed myofibers is associated with alterations in ECM and trophic factors. (A)** Histogram showing the relative proliferation of MPCs cultured in the presence of conditioned media from cultured intact or collapsed myofibers. Results were obtained from four different preparations of conditioned media that were exposed to four separate primary MPC cell lines. **(B)** Graph showing the qPCR quantification of IGF-1 relative mRNA expression. Results were obtained from four independent cultured intact and damaged myofiber preparations isolated from four different mice. **(C)** Histograms showing qPCR relative mRNA expression of the basal lamina genes coding for collagen VI, laminin, fibronectin, and collagen IV. The results compare cultured intact and collapsed myofibers from four different myofiber preparations to the contralateral muscle isolated from separate mice. *P < 0.05, **P < 0.01, ***P < 0.0001, and ****P < 0.00001 for the proliferation and qPCR assays. All results are expressed as means ± SEM. **(D,E)** Representative micrographs of fibronectin immunostaining in cultured intact and collapsed myofibers **(D)** and in the skeletal muscle of WT and *mdx* mice **(E). (D,E)** Fibronectin staining in cells at the periphery (arrowheads), at the basal lamina (arrows), and in the cytoplasm of the collapsed/damaged myofibers (asterisk) can be seen.

The satellite cell microenvironment is profoundly altered following muscle damage and repair [43]. We first determined whether trophic factors secreted by intact or degenerating myofibers can influence MPC proliferation *in vitro* by performing a proliferation assay using MPCs grown in the presence of conditioned media from intact or collapsed myofibers. Our results showed that MPC proliferation increases slightly but significantly (approximately 13.4%; *P* < 0.0239) when MPCs are grown in conditioned media from collapsed myofibers (Figure 5A). Since IGF-1 is a strong mitotic factor that can be produced by skeletal muscle cells [44], we measured IGF-1 expression in intact and collapsed myofibers and detected a significant 2.4-fold (*P* < 0.0002) increase in expression in the collapsed myofibers (Figure 5B). These findings suggested that, under these experimental conditions, factors secreted by myofibers can have a significant impact on MPC proliferation.

We then determined whether the expression of recognized basal lamina components is altered in cultured collapsed myofibers compared to intact myofibers. A qPCR gene analysis showed that collagen VI, laminin, and fibronectin, but not collagen IV, expression are significantly higher in collapsed myofibers than in intact myofibers (Figure 5C). Since fibronectin expression increased the most (approximately 224-fold; *P* < 0.0001) and is known to influence MPC adherence/proliferation, we immunostained (anti-fibronectin) individual cultured intact and collapsed myofibers as well as the skeletal of muscle of *mdx* mice. As expected, fibronectin was detected at the basal lamina of intact myofibers (arrows). We also observed strong staining around cells (arrowheads) located at the basal lamina as well as irregular staining in the cytoplasm (asterisk) of collapsed myofibers (Figure 5D). These observations were corroborated in the skeletal muscles of WT and *mdx* mice where fibronectin was mainly observed in the basal lamina of the myofibers. Strikingly, we observed strong, irregular fibronectin staining only in the cytoplasm of EBD^+^ myofibers (asterisk), which was consistent with the results obtained with cultured myofibers (Figure 5E).

### Collapsed myofibers increase the stiffness of the myogenic progenitor cell microenvironment and favor the proliferation of myogenic progenitor cells

**Figure 6:**
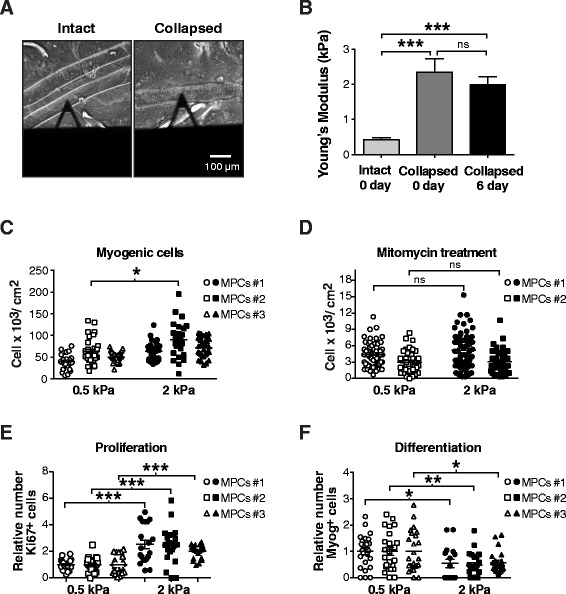
**Collapse of myofibers increases the myogenic progenitor cell microenvironment stiffness and maintains cell proliferation. (A)** Representative phase contrast photomicrographs of freshly isolated intact and collapsed myofibers from 3 WT mice in differentiation medium during AFM measurements. The triangular cantilever is shown. **(B)** Histograms showing the stiffness of isolated intact myofibers (*n* = 27), collapsed myofibers after 0 days of culture (*n* = 26), and collapsed myofibers after 6 days of culture (*n* = 22). The myofiber stiffness values (average of 3 measurements/myofiber) from 3 independent experiments are expressed as kPa. ***P < 0.0001 versus intact myofibers on day 0. **(C)** Scatter dot plot showing the number of myogenic progenitor cells (MPCs or myoblasts)/ cm^2^ after 3 days of culture in proliferative medium on the 0.5- and 2-kPa substrates. The results were obtained by counting eight fields/well and three wells/substrate (*n* = 3). **(D)** Scatter dot plot showing the number of MPCs/cm^2^ after 3 days of culture in proliferative medium on the 0.5- or 2-kPa substrates following treatment with mitomycin. Results were obtained by counting 15 fields/well and 4 wells/substrate (*n* = 2). **(E)** Scatter dot plot showing the percentage of Ki67^+^ MPCs after 3 days of culture in proliferative medium on the 0.5- or 2-kPa substrates. Results were obtained by counting eight fields/well and three wells/substrate (*n* = 3). **(F)** Scatter dot plot showing the percentage of Myog^+^ MPCs after 3 days of culture in proliferative medium on the 0.5- and 2-kPa substrates. Results were obtained by counting eight fields/well and three wells/ substrate (*n* = 3). The primary MPC lines were isolated from 3 different mice. Percentages are expressed as relative values of the 0.5-kPa substrate values. *P < 0.05; **P < 0.01; ***P < 0.0001 versus 0.5 kPa. Results are expressed as means ± SEM.

Increasing evidence indicates that changes in the mechanical properties of the microenvironment have a major impact on stem cell [45-49] and satellite cell function and behavior [7,8]. To determine whether the mechanical microenvironment of collapsed myofibers is altered, we measured the stiffness of intact and collapsed myofibers using an AFM-based indentation approach. The AFM cantilever (black triangle) used to quantify the stiffness of intact (freshly isolated) and collapsed (cultured for 6 days) myofibers can be seen in Figure 6A. There was a 4-fold increase in the stiffness of collapsed myofibers compared to freshly isolated myofibers (*P* < 0.0001; Figure 6B). In addition, there was no significant difference between freshly isolated and collapsed myofibers on day 0 compared to collapsed myofibers after 6 days in culture, indicating that myofiber collapse is the main factor contributing to the increase in microenvironment stiffness.

We determined whether the increased proliferation of collapse-associated MPCs resulted from the greater stiffness of the microenvironment by mimicking the stiffness *in vitro* using stiffness-tunable gel substrates. We used polyacrylamide gels [34] to assess the impact of the stiffness of intact and collapsed myofibers (0.5 and 2 kPa, respectively) on primary MPC behavior. Exposing MPCs for 4 days to a 2-kPa substrate promoted higher proliferation than exposing them to a 0.5-kPa substrate (1.5-fold increase; *P* = 0.004; Figure 6C). On the other hand, there was no difference in MPC proliferation after 2 days of culture on the 0.5- and 2-kPa substrates when the MPCs were pre-treated with mitomycin, a DNA replication blocking agent (Figure 6D), indicating that the increased proliferation of MPCs observed on the 2-kPa substrate was not due to cell detachment. In order to determine the impact of stiffness on MPC proliferation and differentiation, we quantified the expression of Ki67, a nuclear proliferation marker, and myogenin (Myog), a differentiation marker [50], in primary MPCs cultured for 3 days on the 0.5- and 2-kPa substrates. The percentage of proliferating Ki67^+^ cells was significantly higher on the 2-kPa substrate than on the 0.5-kPa substrate (2.4-fold, *P* < 0.0001; Figure 6E), while the percentage of differentiating Myog^+^ MPCs was lower on the 2-kPa substrate than on the 0.5-kPa substrate (1.9-fold, *P* < 0.05; Figure 6F). These results indicated that the collapse of myofibers promotes the proliferation of MPCs by increasing myofiber stiffness.

## Discussion

Determining the factors that contribute to the differentiation of satellite cells and their progeny into functional myofibers is essential for understanding skeletal muscle regeneration in aging and for treating muscular pathologies. We studied individual cultured myofibers that collapse *in vitro*, which promotes the proliferation of MPCs [51,52]. Our results revealed that collapsed myofibers are characterized by a loss of viability and cytoskeletal organization. We also showed *in vivo* that cytoskeletal disorganization is a key determinant for degenerative myofibers and is concomitant with the local proliferation of MPCs. We further demonstrated, using AFM-based indentation experiments, that myofiber collapse is associated with an increase in the stiffness of the microenvironment of the MPCs. Lastly, tunable-stiffness substrates made it possible to recapitulate the changes in muscle stem cell behavior observed with collapsed myofibers.

Culturing individual myofibers is a valuable method for exploring satellite cell behavior *in vitro* because it preserves their anatomical niche. It has been used to highlight the asymmetric division and self-renewal of satellites cells induced by the bipolar arrangement of signals arising from subjacent myofibers and the basal lamina [53]. However, a model that reproduces the myofiber damage and alterations to the niche of satellite cells that occur *in vivo* is lacking. In addition, little is known about the stiffness of the satellite cell microenvironment and its impact on muscle regeneration *in vivo*.

Collapsed myofibers have been described by Bischoff [51] as hypercontracted *in vitro*. They are characterized by an extreme shortening of the sarcomeres and an abnormal band pattern. Collapsed myofibers have been observed following extreme eccentric muscle action [54] and in dystrophic muscles [41,42] but, to our knowledge, they have never been investigated *in vitro*. Unlike traditional primary myogenic cultures prepared from enzymatically dissociated whole muscle, individual myofiber cultures make it possible to study satellite cells and their progeny within their anatomical niche under the basal lamina. We used a Myf5-nLacZ reporter gene and Pax7 immunostaining to show that individual collapsed myofibers display a much larger number of MPCs on their periphery after 6 days of culture than intact myofibers. The large variability in the number of Pax7^+^ cells on individual collapsed myofibers was likely due to random collapse during culture. The characterization of their differentiation states using Pax7 and MyoD immunostaining indicated that MPCs from both intact and collapsed fresh myofibers are mainly quiescent since they are in their physiological niche [25,35,36]. However, when they were cultured, a large proportion became activated and adopted a proliferative state, particularly in collapsed myofibers, indicating that a loss of integrity of the satellite cell substrate influences their activation and favors the proliferation of their progenies.

Sarcolemma disruption and myofiber degeneration are common features of the muscle regeneration process. Previous studies have shown that degenerative and necrotic myofibers are permeable to EBD (EBD^+^) [39]. In the present study, local clusters of proliferative MPCs were closely associated with EBD^+^ myofibers, which was not the case with undamaged EBD^−^ myofibers. Based on phalloidin staining, the EBD^+^ myofibers in *mdx* mice displayed a disorganized cytoskeleton, which could be categorized according to their level of organization. Interestingly, the number of MPCs was proportional to the disorganization state of the actin cytoskeleton, suggesting that that various states may be correlated with the sequence of myofiber degeneration, where the complete loss of actin staining represents the ultimate state. We also showed that α-actinin, a cytoskeletal protein that is essential for the stability of the contractile apparatus responsible for the transmission of the force generated to the extracellular matrix through integrins [37,55-57], is also disorganized in the EBD^+^ myofibers, as shown by the lack of periodicity typical of structured sarcomeres. This suggested that α-actinin disorganization may precede F-actin disorganization, since the organization of the F-actin of a proportion of EBD^+^ myofibers was similar to that of undamaged myofibers.

Since myofiber collapse or shortening appeared to be a key for MPC activation/proliferation, we determined whether MPC proliferation is affected by traumatic muscle shortening. We used a tenotomy model to show that the number of MPCs is significantly higher in tenotomized than in contralateral control muscles. This increase in MPC proliferation confirmed previous findings with respect to [^3^H]thymidine incorporation showing that TA tenotomy promotes the proliferation of stromal and myogenic cells [58]. Unlike *mdx* mice in which MPCs proliferate locally around EBD^+^ myofibers, there were virtually no EBD^+^ myofibers in the tenotomized muscles, and the MPCs were rather scattered throughout the tissue, with a few clusters of MPCs. Despite the absence of degenerating EBD^+^ myofibers, the vast majority of myofibers displayed generalized disorganization, as shown by the loss of width uniformity. In addition, F-actin and α-actinin staining revealed that most of the myofibers had a disorganized cytoskeleton as shown by the lack of periodicity. As such, in addition to collapsed necrotic myofibers, the physiological loss of myofiber tension *in vivo* appeared to be sufficient to induce the activation and proliferation of MPCs *in vivo*.

Changes in the satellite cell niche microenvironment directly impact MPC activities [1,3,43]. This is based on a number of seminal studies showing that Notch, IGF-1, FGF2, Wnt, and TGFβ are involved in the MPC niche and the alteration of MPC activity (reviewed in [59]). Likewise, M-cadherin, a highly expressed cell adhesion molecule in satellite cells, is also involved in MPC activation [60,61], attachment, and fusion during prenatal myogenesis [62]. In the present study, we showed that trophic factors produced by collapsed/damaged myofibers and their MPCs favor MPC proliferation *in vitro*. In fact, IGF-1 expression was higher in collapsed myofibers, which corroborated previous reports that IGF-1 secretion increases in differentiated skeletal muscle cells undergoing biomechanical stimulation (stretching) [44]. Furthermore, Wnt signaling, which dictates the fate of satellite cells by controlling their expansion, is highly dependent on the binding of the ECM glycoprotein fibronectin to syndecan-4 [63]. Interestingly, fibronectin, which plays an important role during wound healing [64] and muscle regeneration [65,66], was highly expressed in damaged myofibers both *in vitro* and *in vivo*. Altogether, our findings highlight the complex nature of the interactions between biochemical factors and the ECM in the MPC microenvironment. This was especially true for MPCs cultured on plastic petri dishes, which are dissociated from their biochemical and biomechanical microenvironments and which invariably lose their stemness and regenerative potential [19,20].

A previous study using AFM-based indentation experiments to determine the stiffness value of skeletal muscle reported a Young’s modulus of approximately 12 kPa [67]. However, this study used skeletal muscle slices composed of multiple structures such as ECM layers that can have an impact on elastic properties. Myofiber bundles are four times stiffer when ECM components are present than individual myofibers that have been stripped of their ECM [9]. Moreover, absolute stiffness values determined by AFM indentation strongly depend on the experimental parameters, model, and parameters used to analyze the results [68].

Interestingly, our AFM-based experiments with individual myofibers revealed that the stiffness values of intact myofibers are four times lower than those of collapsed myofibers (0.5 versus 2.0 kPa). When the stiffness values were recapitulated in gel substrates, the 2-kPa environment promoted the proliferation of MPCs and inhibited their differentiation, maintaining MPCs in a proliferative state. This was in agreement with previous work showing that MPC proliferation is strongly influenced by elasticity while their differentiation is modulated by the protein coating [7]. Studying MPC behavior is of the utmost importance in such contexts since the mechanical microenvironment of MPCs also has a direct impact on their regenerative potential following transplantation [8]. For example, the engraftment scores of MPCs grown on a 12-kPa substrate for 1 week are significantly higher than those of MPCs grown on 2-, 42-, and 1 × 10^6^-kPa substrates, which correspond to brain, cartilage, and plastic petri dish stiffnesses, respectively [8]. While stiffness appears to be a major contributor to the modulation of satellite cell and MPC behavior, further studies will be required to understand the cross-talk between the matrix (mechanotransduction) and the cytokine/growth factors in the microenvironment of degenerative/regenerative myofibers.

## Conclusions

Our results provided new evidence that minor biomechanical alterations in the microenvironment, such as stiffness, are sufficient to directly impact the proliferative and differentiation capacities of muscle stem cells. In essence, they reinforce the role of microenvironmental cues such as mechanical constraints and local factors in MPC behavior during physiological muscle repair, muscle regenerative disorders, and aging.

## Additional file

Additional file 1: Figure S1. The proportion of collapsed myofibers increases as a function of time in culture. Graph displaying the percentage of collapsed myofibers over a period of 6 days in culture (*n* = 5). Over 80% of the myofibers were collapsed on day 6.

## Abbreviations

BF: biceps femoris
ECM: extracellular matrix
EDL: extensor digitorum longus
kPa: kilo Pascal
MPCs: myogenic progenitor cells
TA: tibialis anterior
