# Erratum to: Increased microenvironment stiffness in damaged myofibers promotes myogenic progenitor cell proliferation

**DOI:** 10.1186/s13395-015-0053-7

**Published:** 2015-09-17

**Authors:** Frédéric Trensz, Fabrice Lucien, Vanessa Couture, Thomas Söllrald, Geneviève Drouin, André-Jean Rouleau, Claire M. Dubois, Michel Grandbois, Gregory Lacraz, Guillaume Grenier

**Affiliations:** Research Centre of the Centre Hospitalier de l’Université de Sherbrooke (CRCHUS), Université de Sherbrooke, Sherbrooke, QC Canada; Department of Electrical and Computer Engineering, Faculty of Engineering, Université de Sherbrooke, Sherbrooke, QC Canada; Department of Pediatrics, Faculty of Medicine, Université de Sherbrooke, Sherbrooke, QC Canada; Department of Pharmacology, Faculty of Medicine, Université de Sherbrooke, Sherbrooke, QC Canada; Department of Orthopedic Surgery, Faculty of Medicine, Université de Sherbrooke, 3001–12th Avenue North, Sherbrooke, J1H 5N4 QC Canada; New address: Hubrecht Institute, University Medical Center Utrecht, Utrecht, The Netherlands

## Text

Professor Claire Dubois was missing from our original authors list. This unfortunate mistake is now rectified and her valuable contribution acknowledged by adding her name in this updated authors list.
