# Erratum to: Increased microenvironment stiffness in damaged myofibers promotes myogenic progenitor cell proliferation

**DOI:** 10.1186/s13395-016-0109-3

**Published:** 2016-10-31

**Authors:** Frédéric Trensz, Fabrice Lucien, Vanessa Couture, Thomas Söllradl, Geneviève Drouin, André-Jean Rouleau, Michel Grandbois, Gregory Lacraz, Guillaume Grenier

**Affiliations:** 1 Research Centre of the Centre Hospitalier de l’Université de Sherbrooke (CRCHUS), Université de Sherbrooke, Sherbrooke, QC Canada; 2 Department of Electrical and Computer Engineering, Faculty of Engineering, Université de Sherbrooke, Sherbrooke, QC Canada; 3 Department of Pharmacology, Faculty of Medicine, Université de Sherbrooke, Sherbrooke, QC Canada; 4 Department of Orthopedic Surgery, Faculty of Medicine, Université de Sherbrooke, 3001-12th Avenue North, Sherbrooke, J1H 5N4 QC Canada; 5 New address: Hubrecht Institute, University Medical Center Utrecht, Utrecht, The Netherlands

## Erratum

Following publication of the original article [1] it was brought to our attention that the fourth author’s surname had been misspelt. The author name was incorrectly written as Thomas Söllrald. However, the correct name should be written as Thomas Söllradl.
