# Supplementary figures and images for: Increased microenvironment stiffness in damaged myofibers promotes myogenic progenitor cell proliferation
Source: Skelet Muscle. 2015 Feb 17;5:5. doi: 10.1186/s13395-015-0030-1 (PMC4343274; doi:10.1186/s13395-015-0030-1)

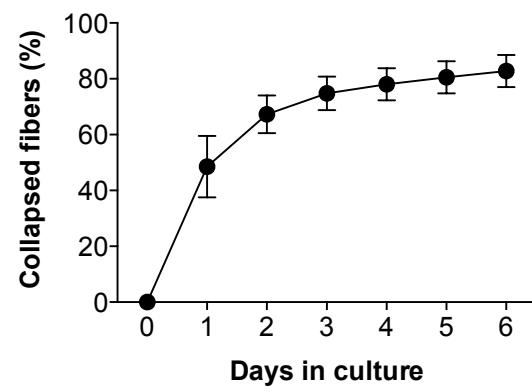

**Figure S1**

Supplement: Additional file 1: Figure S1. — The proportion of collapsed myofibers increases as a function of time in culture. Graph displaying the percentage of collapsed myofibers over a period of 6 days in culture (n = 5). Over 80% of the myofibers were collapsed on day 6. [file 13395_2015_30_MOESM1_ESM.pdf]
